# *rac*-(*E*,*trans*)-4-Bromo-10,10-dimethyl-9,11-dioxabi­cyclo­[6.3.0]undec-4-ene

**DOI:** 10.1107/S2414314620013024

**Published:** 2020-09-30

**Authors:** Dieter Schollmeyer, Maximilian Heidrich, Heiner Detert

**Affiliations:** a Johannes Gutenberg University Mainz, Department of Chemistry, Duesbergweg 10-14, 55099 Mainz, Germany; Goethe-Universität Frankfurt, Germany

**Keywords:** crystal structure, heterocycles, medium-sized ring, bromine

## Abstract

In the title compound, a cyclo­octene ring in a twist-boat conformation and a dioxolane ring with a distorted envelope conformation are annulated in a *trans* configuration. Alternating strands of single enanti­omers build up the crystal. Within the strands, the mol­ecules are connected by weak C—H⋯O hydrogen bonds.

## Abstract 2

In the title compound, C_11_H_17_BrO_2_, a cyclo­octene ring in a twist-boat conformation and a dioxolane ring with a distorted envelope conformation are annulated in a *trans* configuration. Alternating strands of single enanti­omers build up the crystal. Within the strands, the mol­ecules are connected by weak C—H⋯O hydrogen bonds.

## Graphical abstract

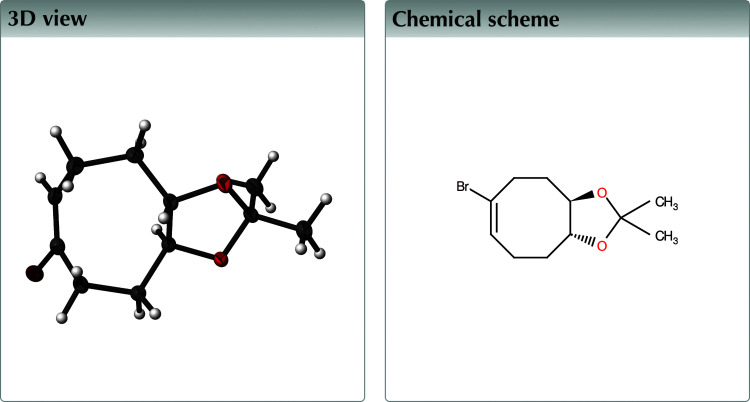

## Structure description

**Figure 1:**
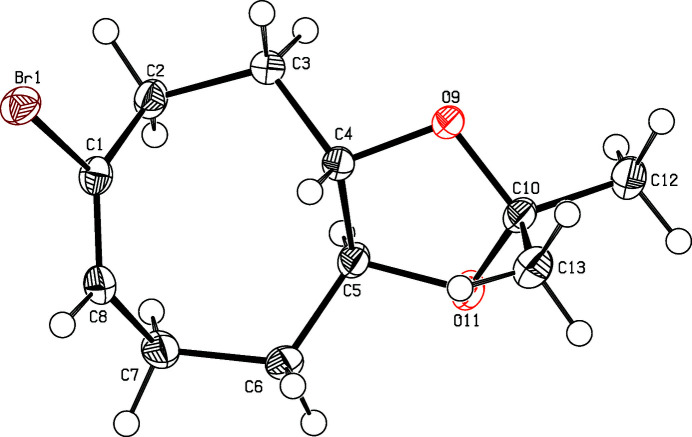
Perspective view of the title compound. Displacement ellipsoids are drawn at the 50% probability level.

**Figure 2:**
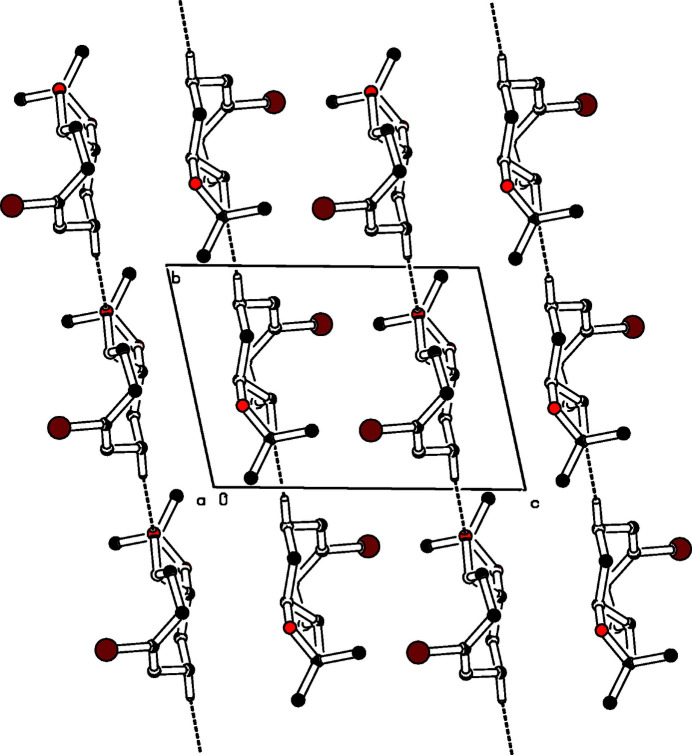
Partial packing diagram of the title compound, viewed along the *a* axis. H atoms not involved in C—H⋯O contacts are omitted.

The title compound (Fig. 1 ▸) crystallizes as a racemic mixture of *R*,*R*- and *S*,*S*-enanti­omers forming strands of identical enanti­omers along the *b*-axis direction (Fig. 2 ▸). The mol­ecules in the strands are connected *via* weak C—H⋯O contacts (H7*B*⋯O9 2.586 Å). A center of inversion relates these mol­ecules with their enanti­omeric counterparts in the parallel strands. The eight-membered ring features a twist-boat conformation. It is annulated to a dioxolane ring in a distorted envelope conformation. Within this envelope, atoms C4, C5, O11, and C10 are essentially coplanar (r.m.s. deviation 0.017 Å) but O9 lies 0.477 (2) Å below the mean plane. The cyclo­octene part has two planar moieties: one is the olefinic part (C2—C1—C8—C7), the other one [planar within 0.034 (2) Å] is composed of the four methyl­ene groups (C3—C2—C7—C6); these planes subtend a dihedral angle of 69.3 (2)°. A *trans*-ethyl­ene bridge (C4,C5) connects the rings; atom C4 lies 0.357 (5) Å above the central plane of the cyclo­octene moiety while C5 is positioned 0.541 (5) Å below this plane.

## Synthesis and crystallization

The title compound was prepared in two steps from 4-bromo-9-oxabi­cyclo­[6.1.0]non-4-ene (Mayer & Meier, 1989 ▸) *via* hydrolysis of the epoxide to the *trans*-diol in alkaline (pH 10) water/dioxane (1/4) [^1^H NMR: 6.02 (*t*, 1H), 3.58 (*m*, 2 H), 3.17 (*s*, 2 H, OH), 2.81 (*ddd*, 1 H), 2.58 (*ddd*, 1 H), 2.0–2.33 (*m*, 4 H), 1.58 (*m*, 12 H); IR: (KBr): 3320, 2918, 1635, 1450, 1430, 1040, 980) and cetalization with 2,2-di­meth­oxy­propane. Alternatively, it may be prepared, more conveniently, from 10,10-dimethyl-9,11-dioxabi­cyclo­[6.3.0]undec-4-ene (Golding *et al.*, 1980 ▸) *via* bromination (Takahashi *et al.*, 2000 ▸) and de­hydro­bromination with 1,8-di­aza­bicyclo­[5.4.0]undec-7-ene (DBU). Procedure: DBU (6 ml) was added to 4,5-di­bromo-10,10-dimethyl-9,11-dioxabi­cyclo­[6.3.0]undec-4-ene (8.92 g, 0.026 mol) in toluene (20 ml) and the mixture was stirred for 72 h. After filtration, the organic layer was washed with water (3 × 20 ml), brine, and dried over MgSO_4_. The solvent was evaporated *in vacuo* and the residue purified by chromatography on silica (cyclo­hexa­ne/ethyl acetate 40/1) to give the title compound as a yellowish oil in 88% yield (5.98 g). Crystallization from ethanol solution yielded colorless crystals, m.p. 313 K.

Spectroscopic data: ^1^H NMR (CDCl_3_): 6.03 (*t*, *J* = 8.2 Hz, 1 H), 3.88 (*m*, 2 H), 2.76 (*ddd*, *J* = 15.1 Hz, *J*′ = 10.4 Hz, *J*′′ = 10.2 Hz), 1 H), 2.54 (*ddd*, *J* = 14.7 Hz, *J* = 6.6 Hz, *J*′′ = 3.9 Hz, 1 H), 2.30 (*m*, 1 H), 2.16 (*m*, 3H), 1.55 (*m*, 2H), 1.38 (*s*, 3H), 1.37 (*s*, 3H). ^13^C NMR: 130.6 (CH), 124.0 (C—Br), 107.7 (O—C—O), 80.7(C—O), 79.9 (C—O), 32.2, 32.0, 29.4, 26.8 (CH_3_), 24.7.

## Refinement

Crystal data, data collection and structure refinement details are summarized in Table 1 ▸.

## Supplementary Material

Crystal structure: contains datablock(s) I, global. DOI: 10.1107/S2414314620013024/bt4098sup1.cif

Structure factors: contains datablock(s) I. DOI: 10.1107/S2414314620013024/bt4098Isup2.hkl

Supporting information file. DOI: 10.1107/S2414314620013024/bt4098Isup3.cml

CCDC reference: 2033919

Additional supporting information:  crystallographic information; 3D view; checkCIF report

## Figures and Tables

**Table 1:** Experimental details

Crystal data	
Chemical formula	C_11_H_17_BrO_2_
*M*_r_	261.15
Crystal system, space group	Triclinic, *P* 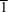
Temperature (K)	120
*a*, *b*, *c* (Å)	7.5770 (6), 7.6838 (6), 10.2038 (8)
α, β, γ (°)	101.843 (6), 90.893 (6), 104.642 (6)
*V* (Å^3^)	561.11 (8)
*Z*	2
Radiation type	Mo *K*α
μ (mm^−1^)	3.64
Crystal size (mm)	0.38 × 0.32 × 0.28

Data collection	
Diffractometer	Stoe IPDS 2T
Absorption correction	Integration (*X-RED32*; Stoe & Cie, 2019 ▸)
*T*_min_, *T*_max_	0.195, 0.460
No. of measured, independent and observed [*I* > 2σ(*I*)] reflections	4770, 2631, 2481
*R*_int_	0.018
(sin θ/λ)_max_ (Å^−1^)	0.658

Refinement	
*R*[*F* ^2^ > 2σ(*F* ^2^)], *wR*(*F* ^2^), *S*	0.034, 0.087, 1.15
No. of reflections	2631
No. of parameters	129
H-atom treatment	H-atom parameters constrained
Δρ_max_, Δρ_min_ (e Å^−3^)	0.73, −0.47

Computer programs: *X-AREA WinXpose*, *Recipe* and *Integrate* (Stoe & Cie, 2019 ▸), *SIR2004* (Burla *et al.*, 2005 ▸), *SHELXL2018/3* (Sheldrick, 2015 ▸) and *PLATON* (Spek, 2020 ▸).

## full crystallographic data

### Crystal data

**Table:**

C_11_H_17_BrO_2_	*F*(000) = 268
*M**_r_* = 261.15	*D*_x_ = 1.546 Mg m^−^^3^
Triclinic, *P*1	Melting point: 313 K
*a* = 7.5770 (6) Å	Mo *K*α radiation, λ = 0.71073 Å
*b* = 7.6838 (6) Å	Cell parameters from 10380 reflections
*c* = 10.2038 (8) Å	θ = 2.8–28.4°
α = 101.843 (6)°	µ = 3.64 mm^−^^1^
β = 90.893 (6)°	*T* = 120 K
γ = 104.642 (6)°	Block, colourless
*V* = 561.11 (8) Å^3^	0.38 × 0.32 × 0.28 mm
*Z* = 2	

### Data collection

**Table:**

Stoe IPDS 2T diffractometer	2631 independent reflections
Radiation source: sealed X-ray tube, 12 x 0.4 mm long-fine focus	2481 reflections with *I* > 2σ(*I*)
Detector resolution: 6.67 pixels mm^-1^	*R*_int_ = 0.018
rotation method, ω scans	θ_max_ = 27.9°, θ_min_ = 2.8°
Absorption correction: integration (X-RED32; Stoe & Cie, 2019)	*h* = −9→9
*T*_min_ = 0.195, *T*_max_ = 0.460	*k* = −9→10
4770 measured reflections	*l* = −13→13

### Refinement

**Table:**

Refinement on *F*^2^	Primary atom site location: dual
Least-squares matrix: full	Hydrogen site location: inferred from neighbouring sites
*R*[*F*^2^ > 2σ(*F*^2^)] = 0.034	H-atom parameters constrained
*wR*(*F*^2^) = 0.087	*w* = 1/[σ^2^(*F*_o_^2^) + (0.0141*P*)^2^ + 2.0148*P*] where *P* = (*F*_o_^2^ + 2*F*_c_^2^)/3
*S* = 1.15	(Δ/σ)_max_ < 0.001
2631 reflections	Δρ_max_ = 0.73 e Å^−^^3^
129 parameters	Δρ_min_ = −0.47 e Å^−^^3^
0 restraints	

### Special details

**Table:**

Geometry. All esds (except the esd in the dihedral angle between two l.s. planes) are estimated using the full covariance matrix. The cell esds are taken into account individually in the estimation of esds in distances, angles and torsion angles; correlations between esds in cell parameters are only used when they are defined by crystal symmetry. An approximate (isotropic) treatment of cell esds is used for estimating esds involving l.s. planes.
Refinement. Hydrogen atoms were placed at calculated positions and were refined in the riding-model approximation with C–H ranging from 0.95 Å to 1.00 Å, and with *U*_iso_(H) = 1.5 *U*_eq_(C_methyl_) or *U*_iso_(H) = 1.2 *U*_eq_(C) for the remaining H atoms.

### Fractional atomic coordinates and isotropic or equivalent isotropic displacement parameters (Å^2^)

**Table:**

	*x*	*y*	*z*	*U*_iso_*/*U*_eq_	
Br1	0.69071 (4)	0.73859 (5)	0.45594 (3)	0.02384 (10)	
C1	0.5268 (4)	0.7199 (4)	0.3056 (3)	0.0190 (6)	
C2	0.5275 (4)	0.5685 (4)	0.1873 (3)	0.0213 (6)	
H2A	0.653108	0.553370	0.179600	0.026*	
H2B	0.491553	0.603426	0.104621	0.026*	
C3	0.3971 (4)	0.3848 (4)	0.1982 (3)	0.0202 (6)	
H3A	0.383050	0.297930	0.110069	0.024*	
H3B	0.453460	0.332706	0.263967	0.024*	
C4	0.2085 (4)	0.3970 (4)	0.2404 (3)	0.0186 (6)	
H4	0.217565	0.462069	0.336747	0.022*	
C5	0.0995 (4)	0.4826 (4)	0.1559 (3)	0.0191 (6)	
H5	0.143866	0.470280	0.063509	0.023*	
C6	0.0962 (4)	0.6811 (4)	0.2095 (3)	0.0216 (6)	
H6A	0.059598	0.693629	0.302984	0.026*	
H6B	0.001262	0.708981	0.155601	0.026*	
C7	0.2785 (4)	0.8259 (4)	0.2082 (3)	0.0243 (7)	
H7A	0.329199	0.797994	0.119793	0.029*	
H7B	0.254789	0.948843	0.219350	0.029*	
C8	0.4184 (4)	0.8321 (4)	0.3168 (3)	0.0219 (6)	
H8	0.429359	0.921776	0.398046	0.026*	
O9	0.0976 (3)	0.2121 (3)	0.2245 (2)	0.0214 (5)	
C10	−0.0876 (4)	0.2202 (4)	0.2147 (3)	0.0208 (6)	
O11	−0.0850 (3)	0.3686 (3)	0.1490 (3)	0.0252 (5)	
C12	−0.1986 (4)	0.0422 (5)	0.1258 (4)	0.0260 (7)	
H12A	−0.149514	0.026435	0.037059	0.039*	
H12B	−0.326342	0.046279	0.116452	0.039*	
H12C	−0.191901	−0.061399	0.166112	0.039*	
C13	−0.1592 (5)	0.2577 (5)	0.3534 (4)	0.0265 (7)	
H13A	−0.081067	0.372480	0.407651	0.040*	
H13B	−0.158069	0.155628	0.397053	0.040*	
H13C	−0.284551	0.269087	0.344726	0.040*	

### Atomic displacement parameters (Å^2^)

**Table:**

	*U*^11^	*U*^22^	*U*^33^	*U*^12^	*U*^13^	*U*^23^
Br1	0.02067 (16)	0.02777 (17)	0.02333 (17)	0.00805 (12)	−0.00350 (11)	0.00432 (12)
C1	0.0179 (14)	0.0207 (14)	0.0172 (14)	0.0025 (11)	−0.0001 (11)	0.0049 (11)
C2	0.0169 (14)	0.0223 (15)	0.0221 (15)	0.0023 (11)	0.0036 (11)	0.0026 (12)
C3	0.0181 (14)	0.0179 (14)	0.0245 (15)	0.0050 (11)	0.0011 (11)	0.0042 (12)
C4	0.0162 (14)	0.0169 (14)	0.0228 (15)	0.0034 (11)	−0.0005 (11)	0.0056 (11)
C5	0.0146 (13)	0.0196 (14)	0.0234 (15)	0.0029 (11)	−0.0011 (11)	0.0073 (12)
C6	0.0194 (14)	0.0219 (15)	0.0249 (16)	0.0077 (12)	−0.0029 (12)	0.0055 (12)
C7	0.0242 (16)	0.0199 (15)	0.0290 (17)	0.0057 (12)	−0.0057 (13)	0.0065 (13)
C8	0.0198 (15)	0.0172 (14)	0.0253 (16)	0.0014 (11)	−0.0019 (12)	0.0019 (12)
O9	0.0145 (10)	0.0178 (10)	0.0331 (13)	0.0040 (8)	0.0007 (9)	0.0088 (9)
C10	0.0142 (13)	0.0214 (15)	0.0281 (16)	0.0042 (11)	−0.0002 (11)	0.0088 (12)
O11	0.0159 (11)	0.0232 (11)	0.0375 (14)	0.0019 (9)	−0.0047 (9)	0.0136 (10)
C12	0.0197 (15)	0.0251 (16)	0.0309 (18)	0.0017 (12)	0.0003 (13)	0.0064 (13)
C13	0.0199 (15)	0.0303 (17)	0.0288 (17)	0.0058 (13)	0.0034 (13)	0.0065 (14)

### Geometric parameters (Å, º)

**Table:**

Br1—C1	1.917 (3)	C6—H6A	0.9900
C1—C8	1.324 (4)	C6—H6B	0.9900
C1—C2	1.496 (4)	C7—C8	1.507 (4)
C2—C3	1.532 (4)	C7—H7A	0.9900
C2—H2A	0.9900	C7—H7B	0.9900
C2—H2B	0.9900	C8—H8	0.9500
C3—C4	1.519 (4)	O9—C10	1.423 (4)
C3—H3A	0.9900	C10—O11	1.433 (4)
C3—H3B	0.9900	C10—C12	1.512 (5)
C4—O9	1.432 (4)	C10—C13	1.521 (5)
C4—C5	1.531 (4)	C12—H12A	0.9800
C4—H4	1.0000	C12—H12B	0.9800
C5—O11	1.441 (4)	C12—H12C	0.9800
C5—C6	1.518 (4)	C13—H13A	0.9800
C5—H5	1.0000	C13—H13B	0.9800
C6—C7	1.541 (4)	C13—H13C	0.9800
			
C8—C1—C2	125.9 (3)	C7—C6—H6B	108.5
C8—C1—Br1	118.7 (2)	H6A—C6—H6B	107.5
C2—C1—Br1	115.3 (2)	C8—C7—C6	113.1 (3)
C1—C2—C3	112.4 (3)	C8—C7—H7A	109.0
C1—C2—H2A	109.1	C6—C7—H7A	109.0
C3—C2—H2A	109.1	C8—C7—H7B	109.0
C1—C2—H2B	109.1	C6—C7—H7B	109.0
C3—C2—H2B	109.1	H7A—C7—H7B	107.8
H2A—C2—H2B	107.9	C1—C8—C7	123.8 (3)
C4—C3—C2	114.7 (3)	C1—C8—H8	118.1
C4—C3—H3A	108.6	C7—C8—H8	118.1
C2—C3—H3A	108.6	C10—O9—C4	106.7 (2)
C4—C3—H3B	108.6	O9—C10—O11	105.1 (2)
C2—C3—H3B	108.6	O9—C10—C12	108.3 (3)
H3A—C3—H3B	107.6	O11—C10—C12	108.9 (3)
O9—C4—C3	107.0 (2)	O9—C10—C13	110.8 (3)
O9—C4—C5	103.2 (2)	O11—C10—C13	110.8 (3)
C3—C4—C5	117.5 (3)	C12—C10—C13	112.7 (3)
O9—C4—H4	109.6	C10—O11—C5	109.5 (2)
C3—C4—H4	109.6	C10—C12—H12A	109.5
C5—C4—H4	109.6	C10—C12—H12B	109.5
O11—C5—C6	107.9 (2)	H12A—C12—H12B	109.5
O11—C5—C4	103.7 (2)	C10—C12—H12C	109.5
C6—C5—C4	117.6 (3)	H12A—C12—H12C	109.5
O11—C5—H5	109.1	H12B—C12—H12C	109.5
C6—C5—H5	109.1	C10—C13—H13A	109.5
C4—C5—H5	109.1	C10—C13—H13B	109.5
C5—C6—C7	115.2 (3)	H13A—C13—H13B	109.5
C5—C6—H6A	108.5	C10—C13—H13C	109.5
C7—C6—H6A	108.5	H13A—C13—H13C	109.5
C5—C6—H6B	108.5	H13B—C13—H13C	109.5
			
C8—C1—C2—C3	−90.1 (4)	Br1—C1—C8—C7	−177.4 (2)
Br1—C1—C2—C3	86.3 (3)	C6—C7—C8—C1	83.7 (4)
C1—C2—C3—C4	46.3 (4)	C3—C4—O9—C10	−158.8 (2)
C2—C3—C4—O9	170.9 (3)	C5—C4—O9—C10	−34.3 (3)
C2—C3—C4—C5	55.6 (4)	C4—O9—C10—O11	32.6 (3)
O9—C4—C5—O11	22.7 (3)	C4—O9—C10—C12	148.9 (3)
C3—C4—C5—O11	140.1 (3)	C4—O9—C10—C13	−87.1 (3)
O9—C4—C5—C6	141.8 (3)	O9—C10—O11—C5	−17.2 (3)
C3—C4—C5—C6	−100.8 (3)	C12—C10—O11—C5	−133.1 (3)
O11—C5—C6—C7	−173.1 (3)	C13—C10—O11—C5	102.5 (3)
C4—C5—C6—C7	70.1 (4)	C6—C5—O11—C10	−129.1 (3)
C5—C6—C7—C8	−74.8 (4)	C4—C5—O11—C10	−3.6 (3)
C2—C1—C8—C7	−1.2 (5)		
